# The DNA Binding Property of PML/RARA but Not the Integrity of PML Nuclear Bodies Is Indispensable for Leukemic Transformation

**DOI:** 10.1371/journal.pone.0104906

**Published:** 2014-08-13

**Authors:** Xi Liu, Hao Yuan, Laurent Peres, Saijuan Chen, Zhu Chen, Hugues de The, Jun Zhou, Jun Zhu

**Affiliations:** 1 CNRS-LIA124, Sino-French Research Center for Life Sciences and Genomics, State Key Laboratory of Medical Genomics, Rui-Jin Hospital, Shanghai Jiao Tong University School of Medicine, Shanghai, China; 2 Université de Paris 7/INSERM/CNRS UMR 944/7151, Equipe Labellisée No. 11 Ligue Nationale Contre le Cancer, Hôpital St. Louis, Paris, France; Institute of Genetics and Molecular and Cellular Biology, France

## Abstract

PML/RARA is the oncoprotein driving acute promyelocytic leukemia (APL). It suppresses genes expression by recruitment of a number of transcriptional repressors, resulting in differentiation block and malignant transformation of hematopoietic cells. Here, we found that mice primary hematopoietic progenitor cells (HPCs), transduced by DNA-binding-defective PML/RARA mutants, were deficient in colony formation. Further experiments showed that DNA-binding-defective PML/RARA mutants could not repress the transcription of retinoic acid regulated genes. Intriguingly, there were no significant differences of the micro-speckled intracellular distribution between the mutants and wild-type PML/RARA. Some retinoic acid target genes regulated by PML/RARA are involved in not only differentiation block but also hematopoietic cell self-renewal. Altogether, our data demonstrate that direct DNA-binding is essential for PML/RARA to immortalize hematopoietic cells, while disruption of PML-nuclear body does not seem to be a prerequisite for hematopoietic cell transformation.

## Introduction

Acute promyelocytic leukemia (APL) accounts for about 12–15% of the incidents of acute myeloid leukemia. In over 98% APL patients a specific chromosomal translocation t(15;17) fuses the *PML* gene on chromosome 15 to the *RARA* gene on chromosome 17. The resulting PML/RARA fusion protein is the molecular determinant of the disease [1]. The presence of a strong dimerization/oligomerization domain in the PML moiety makes PML/RARA as a potent repressor at least in part through the ability of PML/RARA homodimers to recruit nuclear receptor corepressors [2]–[9]. The K160 sumoylation site of PML/RARA which allows the recruitment of a potent repressor DAXX is decisive for *ex vivo* cell immortalization/differentiation arrest and *in vivo* APL pathogenesis [10]. The presence of RXR, the RARA heterodimeric partner, extends the binding of PML/RARA to more widely spaced direct repeats than the normal RARA/RXR heterodimers and is pivotal to transformation *ex vivo* [11]–[14]. These distinct properties of the fusion protein cooperate to enforce transcriptional repression. Intriguingly, the treatment with *all trans* retinoic acid (ATRA) induces complete remissions [15], at least in part through reactivation of PML/RARA-dependent transcription and ubiquitin-proteasome dependent degradation of PML/RARA fusion protein [16].

Recent studies have demonstrated that PML/RARA regulates a novel class of genes through interaction with other transcription factors, such as Sp1 and NF-Y, without directly binding to the DNA, defining a gain-of-function for the oncoprotein [17]. PML/RARA was also shown to transactivate the TF promoter through an indirect interaction with an element composed of a GAGC motif and the flanking nucleotides [18]. It is also reported that PML/RARA predominantly targets PU.1-regulated promoters through both protein-protein interaction and DNA binding via retinoic acid response element (RARE) half sites forming complex binding motifs [19]. Yet, any contribution of this indirect and complex repression of target genes by PML/RARA to differentiation arrest and immortalization of primary hematopoietic progenitors remains unknown.

PML is a nuclear matrix protein that organizes PML nuclear bodies (NBs) [20]. NBs accumulate a wide variety of cellular or viral proteins, including key regulators of cell growth or transformation. They have been proposed to be important for senescence induction or growth control [20], [21]. In APL, PML/RARA alters the intranuclear distribution of PML and other NB-associated proteins. The phenomenon has been theoretically attributed to the hypothesis that PML by forming heterodimer with PML/RARA has been tethered to chromatin through DNA binding domain of the fusion protein. Therefore disruption of NBs has been suggested to contribute to APL pathogenesis.

In this study we have investigated the role of direct DNA binding of PML/RARA in cell transformation and demonstrated that the DNA binding property is critical to cell differentiation arrest and immortalization. Meanwhile, we show that the DNA binding domain is not the molecular determinant for PML/RARA intra-cellular distribution and the disruption of PML NBs integrity is not a prerequisite for full cell transformation.

## Results

### DNA binding defective mutants of PML/RARA failed to bind the RARE and activate the RARE-reporter gene

**Figure 1:**
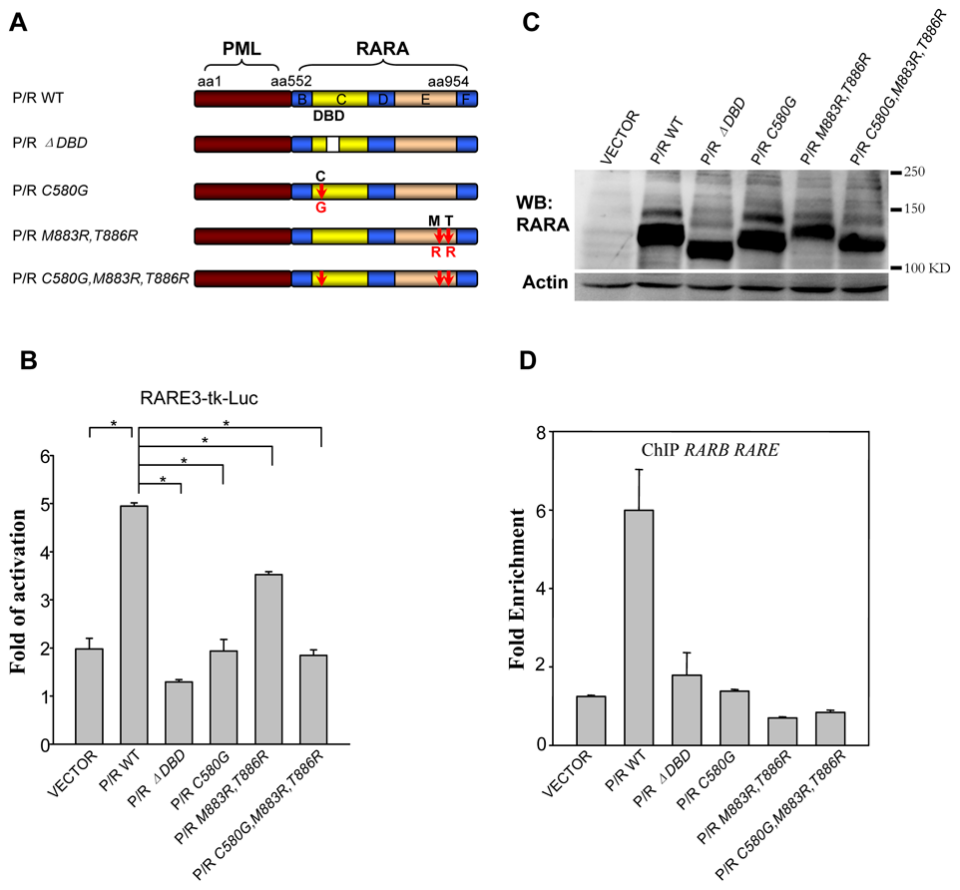
PML/RARA DNA-binding defective mutants failed to bind and activate RARE. (A) Schematic diagram of P/R wild type (WT) and mutants used in the study. B, C, D, E and F, RARA functional regions; DBD, DNA-binding domain. (B) RARE3-tk Luc reporter plasmid and indicated protein expression constructs were transiently cotransfected into 293T cells. ***Renilla*** luciferase plasmid was transfected into cells as internal control. 14 hours after ATRA (10^−6^ M) or ethanol vehicle treatment, transactivation was measured. The number refers to fold of transactivity after ATRA treatment. Mean values were obtained from 3 independent experiments with duplicate wells. Error bars indicate standard deviations of triplicate measurements. *p<0.01. (C) Western blotting analysis of wild type and various mutant PML/RARA proteins with anti-RARA in transiently transfected 293T cells. (D) ChIP analysis of wild type or mutant PML/RARA binding to the endogenous *RARB* promoter in transfected 293T cells, expressed as increase over nonspecific binding. Means ± standard deviation (SD).

The mutation of C88 to G of RARA abrogates its DNA binding and transactivation ability [22]. To demonstrate that the same mutation in PML/RARA (PML/RARA *C580G*) recaptures this property (Figure 1A), luciferase assay of the RA-sensitive reporter gene (RARE-3-Tk-Luc) [23] and ChIP analysis have been conducted. The PML/RARA *C580G* mutant was much less potent than the parental fusion in ligand (ATRA) triggered activation (Figure 1B). Coexpression of RXR can dramatically increase the binding efficiency of PML/RARA. We had previously demonstrated that M883 and T886 of PML/RARA are essential sites for RXR binding [24]. To completely abrogate the DNA binding through heterodimer formation with RXR, *M883R*, *T886R* mutations were introduced in PML/RARA *C580G* mutant (Figure 1A). Like PML/RARA *C580G*, the PML/RARA *C580G*, *M883R*, *T886R* triple mutant displayed very weak transactivation upon ATRA treatment (Figure 1B). The transactivation property of PML/RARA *M883R*, *T886R* is fully consistent with our previous observations (Figure 1B). Note that cells transduced by different PML/RARA mutants expressed similar levels of fusion proteins and had comparable PML/RARA modification adducts (Figure 1C).

In ChIP analysis, as expected, the abundance of enriched genomic DNA fragments containing the *RARB* promoter region precipitated by anti-PML antibody was significantly reduced in transfected 293T cells expressing DNA binding defective mutants, which was consistent with the luciferase results (Figure 1D). No obvious difference was observed in a neighbouring negative control of ChIP assay (data not shown). Similar results were obtained in stable NIH3T3 cells (Figure S1). The same experiment was also performed with a PML/RARA *ΔDBD* mutant lacking entire DNA-binding domain. Unsurprisingly, this mutant displayed similar feature as the DNA binding defective point mutation mutants. (Figure 1D).

### DNA binding property of PML/RARA is required for differentiation arrest and transformation of primary mouse hematopoietic progenitors

**Figure 2:**
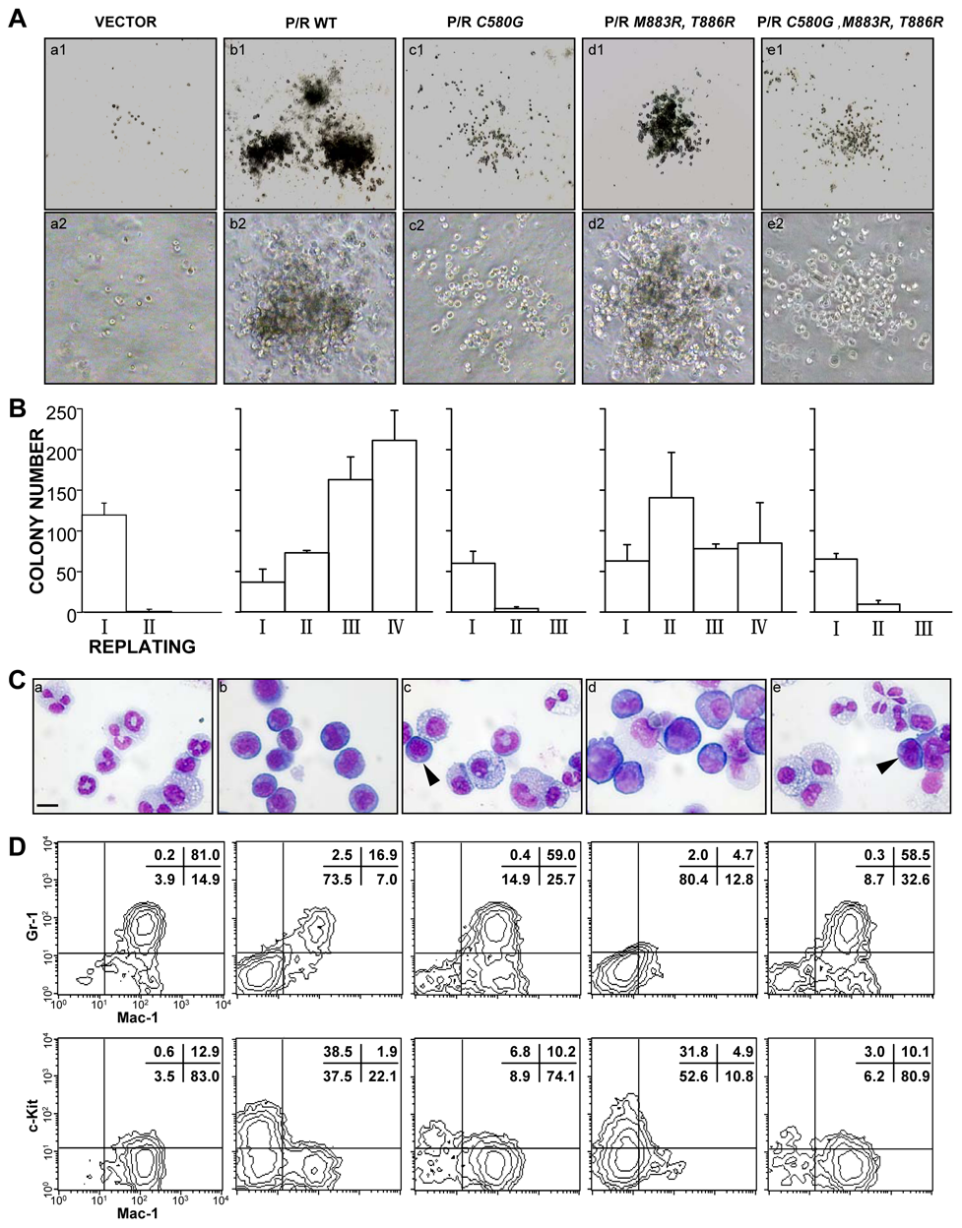
DNA-binding is required for PML/RARA transformation. (A) Typical morphology of the second round colonies generated HPCs transduced with the indicated retroviruses. (B) Serial methylcellulose replating assays of HPCs transduced with the indicated retroviruses. Numbers of colonies in the indicated round plating were obtained from three independent experiments with duplicate dishes. Error bars represent standard deviations. (C) Wright-Giemsa staining of primary hematopoietic precursors transduced with MSCV empty vector, the wild-type or indicated mutant PML/RARA showed histological differentiation of transduced HPCs . Cells were harvested from the first round. Arrowheads indicate transformed bone marrow cells. Representative of three independent experiments. Scale bar, 10 µm. (D) Flow cytometry analysis of the corresponding transduced HPCs using three myeloid differentiation markers, c-Kit, Mac-1, and Gr-1. Representative of three independent experiments.

Previous studies suggested that the RARA DNA binding region of the fusion protein contributed only partially to the differentiation block [25]. We then questioned whether DNA binding properties would be dispensable for PML/RARA-induced transformation of primary hematopoietic progenitors *ex vivo* [26]. The colony formation assay of mouse hematopoietic progenitors was carried out. The cells transduced by PML/RARA could be replated indefinitely in methylcellulose with immature myeloid progenitor morphology. In sharp contrast, those cells transduced by DNA binding defective mutants, PML/RARA *C580G* and PML/RARA *C580G*, *M883R*, *T886R* could not trigger a sharp differentiation arrest and failed to allow indefinite colony formation in the replating assay (Figure 2A and 2B). PML/RARA *C580G* expressing cells were differentiated into both granulocytes and monocytes, as assessed by both MGG staining and myeloid antigens Gr-1 and Mac1 expression analysis (Figure 2C and 2D). PML/RARA *C580G*, *M883R*, *T886R* transduced cells behaved similarly to the PML/RARA *C580G* ones. Nevertheless, PML/RARA *M883R*, *T886R*-transformed cells underwent a sharp differentiation arrest and became immortal as previously reported [14] (Figure 2A to 2D). Again, cells transduced by different PML/RARA mutants expressed similar level of fusion protein and comparable PML/RARA sumoylation patterns were present for each mutant (data not shown).

### The disruption of PML nuclear bodies is insufficient for full leukemic transformation of primary mouse hematopoietic progenitors

**Figure 3:**
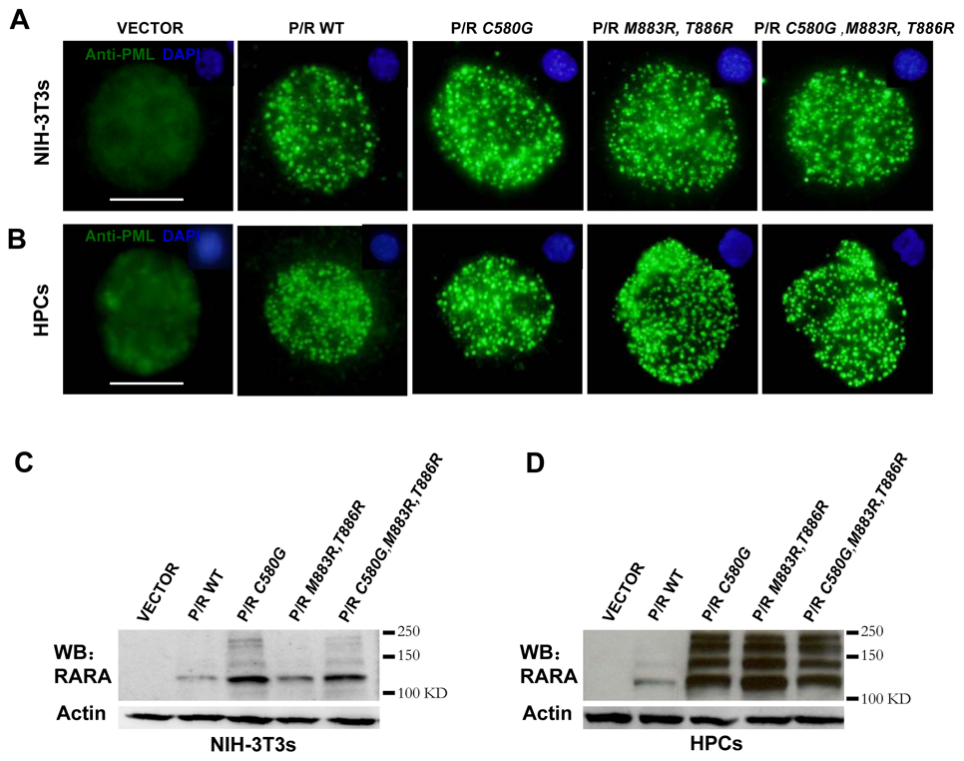
DNA-binding defective PML/RARA mutants disrupt PML nuclear bodies. NIH-3T3 cells (A and C) or mouse HPCs (B and D) were infected with retroviral constructs expressing wild type or the indicated mutant PML/RARA respectively. Immunofluorescence analysis (A and B) was carried out with a rabbit anti-PML specific for PML/RARA to show distribution of wild type or mutant PML/RARA in nucleus, and with DAPI to visualize nuclei (top). Mouse PML was not recognized by the rabbit anti-PML. HPCs were harvested from the first round colonies. Representative of three independent experiments. Scale bar, 10 µm. Western blotting analysis of fusion proteins with anti-RARA in transduced NIH-3T3 cells (C) or mouse HPCs (D).

PML nuclear bodies play an important role in the control of apoptosis or senescence and are disrupted upon PML/RARA expression [27], [28]. To investigate whether the integrity of PML NBs is tightly linked to DNA binding and cell transformation, an immuno-fluorescence assay has been performed in the cells expressing different PML/RARA mutants. Surprisingly, PML/RARA DNA binding defective mutants disrupted PML NBs both in stably transfected NIH 3T3 cells or transduced mouse hematopoietic progenitors (Figure 3A and 3B). Note the expression of each mutant was comparable as examined by western blot (Figure 3C and 3D). Since rabbit anti-PML antibodies used did not recognize endogenous murine PML, immunofluorescence experiments were re-performed in Hela cells. As expected, while the typical NBs were revealed by double staining of anti-PML and anti-SP100 in control cells, they were destroyed and replaced by PML/RARA specific micro-speckles in PML/RARA mutants transfected cells (Figure S2). These observations therefore imply that disruption of PML NBs is not sufficient for PML/RARA to fully immortalize primary hematopoietic progenitor cells and the DNA binding is not the mechanism through which PML NBs are disrupted by PML/RARA.

### Expression of endogenous ATRA response genes in cells transduced by PML/RARA and DNA binding defective mutants

**Figure 4:**
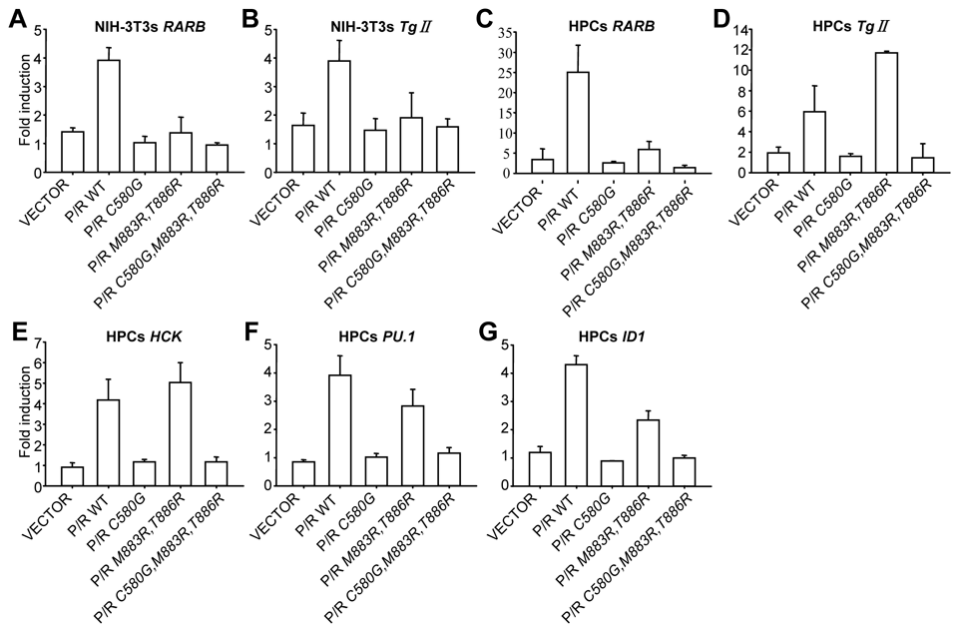
Transcriptional regulation of endogenous target genes by PML/RARA mutants. (A-B) NIH-3T3 cells stably expressing the indicated PML/RARA mutants were treated or not with 10^−6^ M ATRA overnight. *RARB* and *Tg II* gene expression were analyzed by quantitative RT-PCR. (C-G) The indicated mutant PML/RARA-transduced primary hematopoietic progenitors were treated with or without 10^−6^ M ATRA for 14 hours. *RARB*, *Tg II*, *HCK*, *PU.1* and *ID1* gene expression was also analyzed by quantitative real-time PCR. All results from quantitative real-time PCR were normalized to *β-actin*. Results, obtained from three independent experiments, are given as mean ±standard deviations.

Transcriptional repression of ATRA target genes induced by PML/RARA was proposed to underlie the APL pathogenesis [6], [29]. We thus examined whether the DNA binding defective mutants retain the ability to repress endogenous ATRA target genes and impair ATRA response in stably transduced NIH 3T3 cells and primary mouse hematopoietic progenitors by quantitative RT-PCR. We first focused on two types of genes: a well characterized RARA target *Rarb* [23] and one primary PML/RARA target, *transglutaminase type II* (*Tg II*) [30]. Only PML/RARA reproducibly activated the expressions of these two genes in NIH 3T3 cells, with PML/RARA C580G, PML/RARA *M883R*,*T886R* and PML/RARA *C580G*, *M883R*,*T886R* being completely inactive (Figure 4A and 4B). In transduced primary hematopoietic progenitors a clear induction of *Rarb* and *Tg II* expression was observed in response to ATRA administration for both PML/RARA and PML/RARA *M883R*, *T886R* (Figure 4C and 4D). The epigenetic chromatin environment of the PML/RARA binding site(s) within the *Tg II* promoter may significantly differ between fibroblasts and transformed primary hematopoietic progenitors, which could explain why the activation was observed in one system, but not the other. Contrarily to the prevailing model, PML/RARA expression did not induce significant changes in the baseline levels of the two target genes (data not shown).

It is reported that PML/RARA predominantly targets PU.1-regulated promoters through both protein-protein interaction and DNA binding via RARE half sites forming complex binding motifs [19]. We thus examined the transcriptional activation of *PU.1* and *HCK* in the presence of different PML/RARA mutants. The activation of *PU.1* and *HCK* were not detected in transduced NIH 3T3 cells, perhaps due to the absence of hematopoietic related genes expression in fibroblasts (data not shown). In contrast, the expression of *PU.1* and *HCK* were only detected in the hematopoietic progenitors transduced by PML/RARA or PML/RARA *M883R, T886R*, but not in those expressing PML/RARA *C580G* and PML/RARA *C580G, M883R, T886R*, again demonstrating that the inability of DNA binding affects ATRA response of endogenous genes (Figure 4E and 4F).

Finally the expression of *ID1* was tested. To our surprise, PML/RARA and PML/RARA *M883R*, *T886R* were still capable of activating the endogenous *ID1* gene in transduced cells, suggesting that a consensus RARE may exist within the promoter of *ID1* (Figure 4G).

ATRA treatment induced degradation of PML/RARA may remove corepressors and allow induction of target genes expression. No obvious difference of protein degradation was observed between PML/RARA and mutants after ATRA or arsenic trioxide (ATO) treatment (Figure S3), suggesting the protein degradation and corepressors release may not play critical roles on the expression of genes tested.

## Discussion

A combination of PML-mediated RARA homodimerization, enhanced corepressor binding, and repression of ATRA target genes was proposed to trigger the APL-specific differentiation block [6], [29]. Three recently published reports demonstrated that PML/RARA regulates a novel class of target genes such as *ID1* and *TF* through interaction with other transcription factors, without directly binding to the DNA, defining a gain-of-function for the oncoprotein [17], [18]. The contribution of this gain of function of PML/RARA in the transformation of primary hematopoietic progenitor cells was never tested. This work, probing PML/RARA function in primary mouse hematopoietic progenitor cells, demonstrates that the direct DNA binding property is indispensible for the fusion protein to impair differentiation and immortalization of the primary cells *ex vivo*. Hence, reduced DNA-binding affinity likely plays a critical role in the weaker phenotype of the DNA binding defective PML/RARA *C580G* mutant. Our experiments in primary cells also confirm the critical importance of DNA binding previously shown in the U937-differentiation assay [25]. Note that, in the context of RARA-triggered *ex vivo* transformation, mutation in the DNA-binding domain abolished *ex vivo* transformation as well [26], likely reflecting the absence of any dimer formation.

The greater biological effects of PML/RARA, when compared to its defective mutants, do not reflect a higher level of protein expression, as the mutants were usually expressed at higher levels than the parental fusion. Moreover, the pattern of posttranslational modification was identical between the parental and the mutant proteins (Figure 1B, Figure 3C and Figure 3D). We favor the idea that the greater expression of the mutant fusion could result from either its reduced toxicity and/or its greater stability. Both of these features may reflect its lower affinity for DNA, as DNA binding was shown to be a critical determinant of RARA degradation [16].

PML NBs are extrachromosomal nuclear matrix associated structures that contain the PML and numerous partner proteins and are disrupted in APL cells in a treatment-reversible manner. Several pathological states are associated with abnormalities of NBs structures, including transformation, viral infection and neurodegenerative disorders. In the transduced primary cells, the expression of DNA binding defective mutants were able to disrupt NBs while these cells could not be immortalized (Figure 2 and 3), suggesting that the disruption of PML NBs is not sufficient for leukemic transformation. Moreover, the K160 sumoylation site in the PML moiety of PML/RARA was required for efficient immortalization/differentiation arrest *ex vivo* as previously shown [10]. In supporting our observation, the PML NBs were also disrupted in PML/RARA K160R transduced cells.

Our findings provide evidence that direct DNA-binding is indispensible for PML/RARA to transform hematopoietic cells and disruption of PML-nuclear bodies is not sufficient for full cell leukemic transformation.

## Materials and Methods

### Constructs and reagents

P/R *ΔDBD* and P/R *M883R, T886R* mutants were described previously [24], [25]. The P/R *C580G* and P/R *C580G, M883R, T886R* mutants were constructed by site-directed mutagenesis using QuikChange Multi Site-Directed Mutagenesis Kit (Stratagene). Wild type and mutant P/R cDNAs were cloned into the pSG5m eukaryotic expression vector, pMSCV-hygromycin and pMSCV-neomycin retroviral vectors. All mutants were confirmed by DNA sequencing. ***All Trans***-Retinoic Acid (ATRA) (Sigma) was dissolved in ethanol as a stock solution at 1 mM and added to cells at a final concentration of 10^−6^ M. Arsenic trioxide (ATO) (Sigma) was kept as a 10^−3^ M stock solution and added to a 10^−6^ M final concentration.

### Cell lines and mice

293T, NIH-3T3 and Plat-E cells were cultured in DMEM (Invitrogen) supplemented with 10% fetal bovine serum (FBS) (Front). For Plat-E cells, blasticidin (10 µg/ml) and puromycin (1 µg/ml) were added to the culture medium [31]. Cultures were placed in a 37°C humidified incubator with 5% CO2. Wild type C57BL/6 mice were bought bred and maintained under specific pathogen-free conditions in the facility at the Shanghai Jiao Tong University School of Medicine. All experimental manipulations were undertaken in accordance with the National Institute of Health Guide for the Care and Use of Laboratory Animals, with the approval of the Scientific Investigation Board of Shanghai Jiao Tong University School of Medicine.

### Virus production

For virus production, the indicated retroviral constructs were transfected into Plat-E packaging cells using Effectene Transfection Reagent (QIAGEN) according to the manufacturer's recommendations. 48 hours later, virus-containing supernatants were harvested, and stored at −80°C or immediately used for infection of cells.

### Colony-forming cells assays

Colony-forming cells assay was performed as described previously [10], [26]. Briefly, wild type C57BL/6 mice, 6- to 8-week old, were injected with 150 mg/kg 5-fluorouracil (Sigma) via tail vein. Five days later, bone marrow cells were harvested by flushing the femur and tibia of mice with ice-cold RPMI 1640 medium (Invitrogen) containing 1% FBS and penicillin/streptomycin (100 IU/ml, 100 µg/ml, respectively) (Invitrogen). Red blood cells were lysed and removed with Ammonium Chloride solution (Stem cell technologies). Lineage antigen positive (CD5+, CD11b+, CD19+, CD45R+, Ly-6G+, TER119+) cells were depleted by using Mouse Hematopoietic Progenitor Enrichment kit (Stem cell technologies) according to the protocol. Enriched hematopoietic progenitors were suspended in RPMI 1640 medium supplemented with 10% FBS, 0.055 mM β-mercaptoethanol, 0.2 mM L-glutamine (Invitrogen), 10 ng/ml each of mIL-3 and mIL-6, 100 ng/ml mSCF (R&D Systems), and penicillin/streptomycin (100 IU/ml, 100 µg/ml, respectively), at a concentration of 1 × 10^6^/ml, and seeded in 24-well plates. Then hematopoietic progenitors were infected with retrovirus supernatant plus 10 ng/ml each of mIL-3 and mIL-6, 100 ng/ml mSCF, and 4 µg/ml polybrene (Sigma), by centrifugation for 2 hours at 3000 rpm at 32°C. The next day, the cells were reinfected. 12 hours after the second infection, the transduced cells were cultivated in Myelocult 3231 methylcellulose medium (Stem Cell Technologies) supplemented with 10 ng/ml each of mGM-CSF, mIL-3 and mIL-6, 100 ng/ml mSCF, and 1.2 mg/ml G418 for selection. Seven days later, the cells harvested from G418-resistant colonies were either analyzed (western blotting, Wright-Giemsa staining, Flow Cytometry, immunofluorescence, and quantitative real-time PCR) or replated. The cells were serially replated until there were no colonies and cells growth.

### Morphological staining

Mouse bone marrow cells harvested from colonies were cytospun onto slides and stained with Wright-Giemsa staining solution following manufacture's manual. The samples were observed under a light microscope (Olympus).

### Immunophenotype analysis

Transformed cells obtained from colonies were stained with the following antibodies: FITC-conjugated anti-Mac-1, PE-conjugated anti-Gr-1, and APC- conjugated anti-c-Kit (eBioscience). Analysis was performed on a FACScalibur cytometer (Becton Dickinson).

### Immunofluorescence microscopy

Transduced NIH-3T3 cells or mouse bone marrow cells harvested from colonies were cytospun onto slides, sequentially fixed in 4% formaldehyde, permeabilized and washed in phosphatebuffered saline (PBS) containing 0.1% Tween-20. Then cells were immunolabelled with 1∶200 diluted rabbit polyclonal antibodies raised in the lab against human PML, washed, and then relabelled with FITC-coupled anti-rabbit antibody. For nucleus **staining**, immunolabeled cells were incubated with 4′, 6′-diamidino-2-phenylindole (DAPI).

### Immunoblotting

Cells were lysed with laemmli sample buffer (Sigma), and then cell lysates were resolved by 7% SDS-PAGE, and proteins were transferred onto nitrocellulose membrane using the iBlot Dry Blotting System (Invitrogen) according to the user's manual. Western blotting was performed with 1∶1000 diluted rabbit anti-RARA (a gift from Dr Chambon's lab), and rabbit anti-Actin as an internal control. Protein signals were detected with the chemiluminescent substrate SuperSignal West Pico (Pierce Biotechnology).

### Luciferase reporter assay

Cells were transiently cotransfected with indicated plasmids (120 ng expression vector, 40 ng *RARE3-tk-Luc* reporter [23], and 20 ng *Renilla-tk-Luc* vector) using Effectene Transfection Reagent (QIAGEN). Cells were treated with ATRA (10^−6^ M) or ethanol vehicle overnight. Luciferase activities were measured on a luminometer using the Dual Luciferase Reporter Assay Kit (Promega) according to the manufacturer's protocol. Firefly luciferase activities were normalized to the ***Renilla*** luciferase activities to correct for differences in transfection efficiency. Assays were performed in duplicate and standard deviations calculated.

### Quantitative real-time polymerase chain reaction (PCR)

Total cellular RNA samples were extracted with RNeasy mini kit (QIAGEN) following the handbook. The cDNAs were synthesized with reverse transcription system (Promega). The quantitative real-time PCR were performed by a Light-Cycler system (Roche) according to the manufacturer's instructions. The target genes transcription level was normalized to the internal *β-actin*. The primers used are listed as follows: mouse *Tg2*, forward: 5′-CTC ACG TTC GGT GCT GTG-3′, reverse: TCC CTC CTC CAC ATT GTC A; mouse *RARB*, forward:5′-CGG CAT ACT GCT CAA TCC A-3′, reverse: 5′-CAA ACG AAG CAG GGC TTG-3′; mouse *HCK*, forward: 5′-AGC CAC TGC CAA AAC TCA TT-3′, reverse: 5′-GCT CAA TGA AGG CCA TGC-3′; mouse *PU.1*, forward: 5′-GGA GAA GCT GAT GGC TTG G-3′, reverse:5′-CAG GCG AAT CTT TTT CTT GC-3′; mouse *ID1*, forward: 5′-GCG AGA TCA GTG CCT TGG-3′, reverse: 5′-CTC CTG AAG GGC TGG AGT C-3′and mouse *β-actin*, forward: 5′-CTA AGG CCA ACC GTG AAA AG-3′, reverse: 5′-ACC AGA GGC ATA CAG GGA CA-3′.

### ChIP assay

Transiently transfected 293T cells or stably transduced NIH-3T3 cells (infection twice and antibiotic selection for 1∼2 weeks) were cross-linked 1% formaldehyde for 10 minutes at 37°C. Cross-linking was stopped by adding glycine at a final concentration of 125 mM. Cells were washed twice with PBS supplanted with 1 mM PMSF and Protease Inhibitor Cocktail (Roche), then resuspended in swelling buffer (25 mM HEPES, pH7.8, 1.5 mM MgCl2, 10 mM KCl, 0.5%NP-40, 1 mM DTT), and incubated for 10 minutes at 4°C, The solutions were vortexed several times during this period to release nuclei and disperse cell clumps. After centrifugation for 5 minutes at 5000 rpm at 4°C, the nuclei pellet was resuspended in SDS lysis buffer (1% SDS, 10 mM EDTA, and 50 mM Tris-HCl [pH 6.8]), and sonicated to shear DNA using the Bioruptor (Cosmo Bio), at high intensity for 15 minutes with 30-second intervals. Insoluble material was removed by centrifugation for 10 minutes at 4°C. Supernatant was precleared by adding 30 µl Protein G Agarose/Salmon Sperm DNA for 2 hours at 4°C with rotation in ChIP dilution buffer (0.01% SDS, 1.1% Triton-X-100, 1.2 mM EDTA, 16.7 mM Tris-HCl [pH6.8] and 167 mM NaCl) containing protease inhibitors, and then centrifuged to remove agarose. Supernatant fraction was incubated with 30 µl Protein G Agarose/Salmon Sperm DNA and 2.5 µl anti-PML antibody overnight at 4°C with rotation. Protein A garose-antibody/chromation complex were harvested by centrifugation and washed once with low salt buffer (0.1% SDS,1% Triton X-100, 0.5% sodium deoxycholate, 2 mM EDTA, 20 mM Tris-HCl [pH 8], 150 mM NaCl and 1 mM DTT), once with high salt buffer (0.1% SDS, 1% Triton X-100, 2 mM EDTA , 20 mM Tris-HCl [pH 8] and 500 mM NaCl), once with LiCl buffer (25 mM LiCl, 1% IGEPAL CA630, 1% sodium deoxycholate, 1 mM EDTA and 10 mM Tris-HCl [pH 8]) and twice with TE buffer (1 mM EDTA and 20 mM Tris-HCl [pH 8]). Chromatin antibody complexes were eluted with Elution buffer (1% SDS and 0.1 M NaHCO3). Cross-linking was reversed with the addition of NaCl (0.5 M final concentration) and incubation of the eluted samples for 5 hours at 65°C. DNA was extracted with phenol/chloroform, and precipitated by the addition of 0.1 volume of 1 M sodium acetate (pH 5.2), 20 µg glycogen (Fermentas) and 2.5 volumes of ethanol. Precipitated DNA was dissolved in 10 mM Tris-HCl buffer (pH 7.4).

Immunoprecipitated DNA as well as inputs were analyzed by quantitative real-time PCR for genomic sequences from the *RARB* promoter region containing RARE and from the *RARB* exon as negative control region. Quantitative real-time PCR was performed using SYBR Premix Ex Taq (Takara) according to the manufacturer's instructions. Primers used were described previously [17], sense 5′-TTGGGTCATTTGAAGGTTAGCA-3′ and antisense 5′-CACACAGAATGAAAGATTGAATTGC-3′.

## Supporting Information

Figure S1 ChIP analysis of wild type or mutant PML/RARA binding to the endogenous *RARB* promoter (A) and the neighbouring negative control region (B) in stable NIH-3T3 cells. Means ± standard deviation (SD).
(TIF)

Figure S2 Immunofluorescence analysis was carried out with a rabbit anti-PML (green) and a chicken anti-SP100 (red) to show distribution of NBs, and with DAPI to visualize nuclei in Hela cells. While the typical NBs were observed in control cells, they were destroyed and replaced by PML/RARA specific micro-speckles in PML/RARA mutants transfected cells. Note that the endogenous SP100 only co-localized with PML but not PML/RARA.
(TIF)

Figure S3 293T cells transfected with indicated PML/RARA mutants were treated or not with ATRA (A) or arsenic trioxide (ATO) (B) overnight, respectively. Western blots were performed with anti-RARA antibody.
(TIF)
