# Supplementary figures and images for: The DNA Binding Property of PML/RARA but Not the Integrity of PML Nuclear Bodies Is Indispensable for Leukemic Transformation
Source: PLoS One. 2014 Aug 13;9(8):e104906. doi: 10.1371/journal.pone.0104906 (PMC4131979; doi:10.1371/journal.pone.0104906)

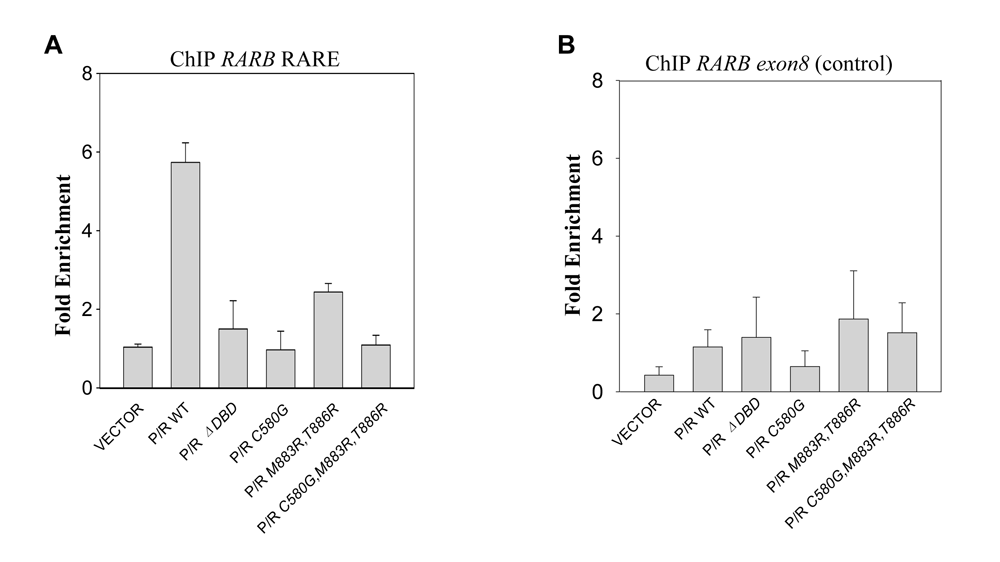

Supplement: Figure S1 — ChIP analysis of wild type or mutant PML/RARA binding to the endogenous RARB promoter (A) and the neighbouring negative control region (B) in stable NIH-3T3 cells. Means ± standard deviation (SD). (TIF) [file pone.0104906.s001.tif]

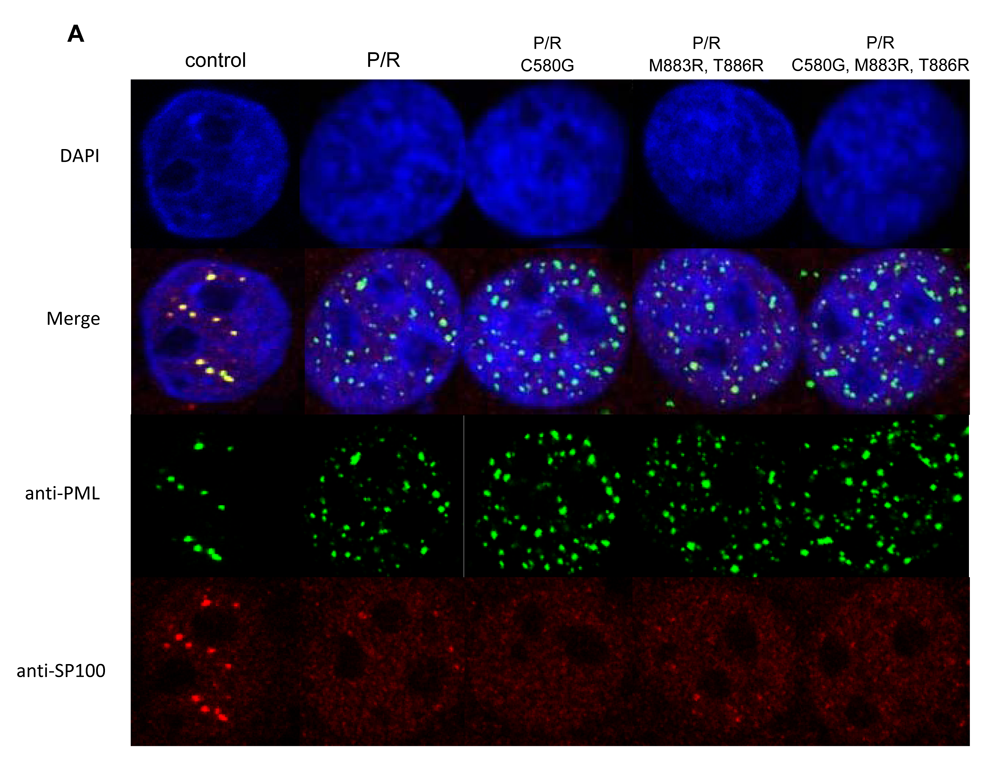

Supplement: Figure S2 — Immunofluorescence analysis was carried out with a rabbit anti-PML (green) and a chicken anti-SP100 (red) to show distribution of NBs, and with DAPI to visualize nuclei in Hela cells. While the typical NBs were observed in control cells, they were destroyed and replaced by PML/RARA specific micro-speckles in PML/RARA mutants transfected cells. Note that the endogenous SP100 only co-localized with PML but not PML/RARA. (TIF) [file pone.0104906.s002.tif]

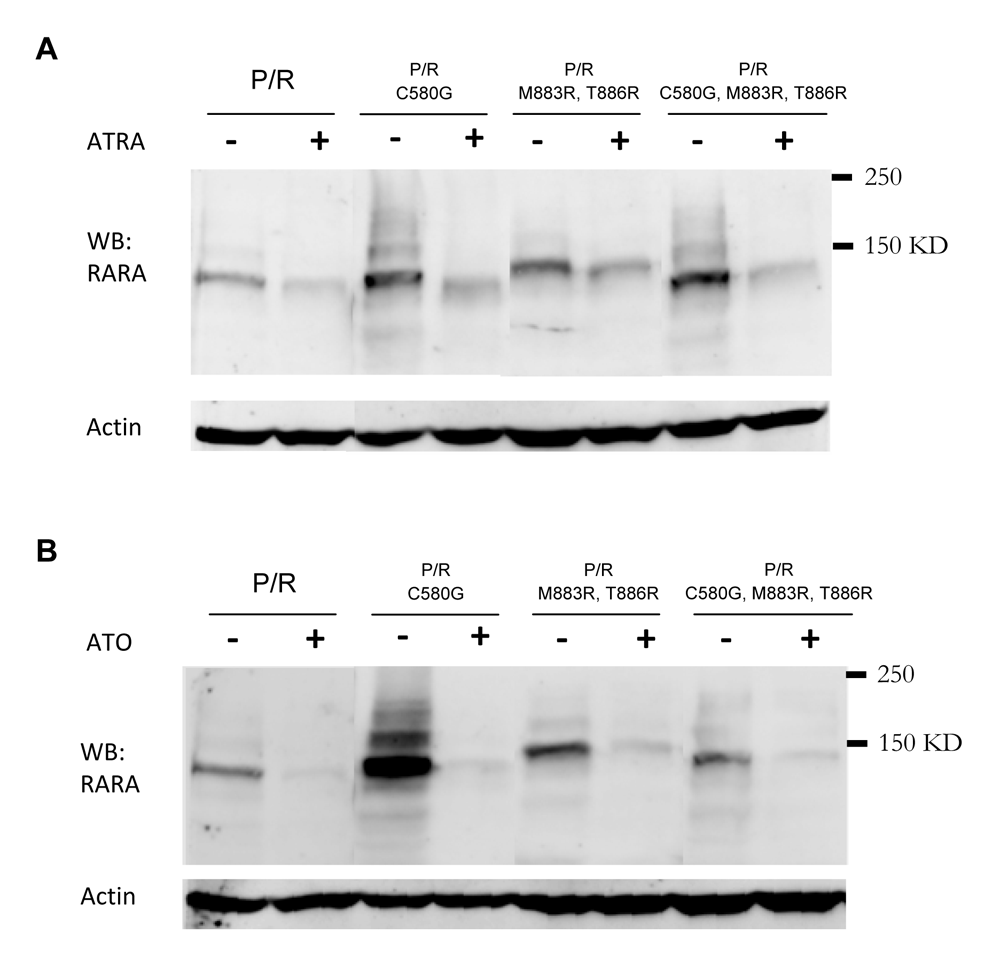

Supplement: Figure S3 — 293T cells transfected with indicated PML/RARA mutants were treated or not with ATRA (A) or arsenic trioxide (ATO) (B) overnight, respectively. Western blots were performed with anti-RARA antibody. (TIF) [file pone.0104906.s003.tif]
